# Case Report: Reproductive evaluation of a Murgese stallion with obstructive azoospermia, accumulation of hyaline material in the ampullae ducts, and corpora amylacea in vesicular glands

**DOI:** 10.3389/fvets.2025.1571637

**Published:** 2025-04-11

**Authors:** Roberta Bucci, Salvatore Parrillo, Monica Probo, Vincenzo Varasano, Anastasia Romano, Augusto Carluccio

**Affiliations:** ^1^Department of Veterinary Medicine, University of Teramo, Teramo, Italy; ^2^Department of Veterinary Medicine and Animal Sciences, University of Milan, Lodi, Italy

**Keywords:** breeding soundness evaluation, alkaline phosphatase, obstructive azoospermia, corpora amylacea, endoscopy, equine andrology

## Abstract

This report aims to present a case of obstructive azoospermia in a stud stallion diagnosed with an alkaline phosphatase (SPAP) assessment. A 20-year-old Murgese stallion is referred for acquired azoospermia. History is negative for reproductive disorders, and clinical examinations and ultrasonography of internal and external genitalia do not reveal significant alterations. Semen collection highlights the absence of spermatozoa in the ejaculate and the urinalysis is negative for spermatozoa. SPAP assay is performed on seminal plasma, with a value of 30 IU/L, compatible with obstructive azoospermia. A biopsy is performed, detecting the presence of complete germ lines in both testes. A resolution is attempted endoscopically, gently insufflating ampullae, with negative results, so the stallion is excluded from breeding. Time afterwards, the stallion dies of natural causes, and necropsy and histopathological analyses are performed. Corpora amylacea are highlighted in both seminal vesicles; the right and left ampullae show ectasic lumen, with the diffuse presence of hyaline material. Ampullae obstruction is an uncommon pathology, which can affect stallions and jacks, generally caused by the accumulation of spermatozoa, but, unfortunately, this case was unresponsive to attempted treatments. Interestingly, to the authors’ knowledge, this is the first report of corpora amylacea in equine stallion sexual glands.

## 1 Introduction

The breeding soundness evaluation (BSE) was introduced in 1975 (1, 2) for the assessment of stud horse fertility and adapted for other species, such as dogs (3), bulls (4), and cats (5). This procedure aims to determine whether a stallion has the mental and physical competence to deliver viable spermatozoa (and no infectious disease) to the female reproductive tract, determining the establishment of pregnancy (6). BSE in stud stallions is recommended at the beginning of each breeding season, before purchase and, particularly, in cases of known or suspected infertility (7). BSE starts with an accurate history, a thorough general clinical examination, and evaluation of the external and internal genitalia. Semen collection and evaluation is also mandatory (8). Currently, ultrasound examinations are routine, alongside physical assessment. Further valuable collateral tests to investigate infertility are hormonal dosages, stimulation tests, testicular biopsies, and cytology (9). Clinical findings set the choice of the most appropriate ancillary analysis, and one of the objectives of this report is to describe the diagnostic process followed in a case of obstructive azoospermia in a stallion.

Azoospermia refers to the complete absence of spermatozoa in the ejaculate (10). This condition is reported to be rare in stallions and can be due to spermatogenesis failure (10, 11). In most cases of suspected azoospermia, ejaculation disorders are found instead (11, 12). Two different events can be involved: the emission, determined by the contraction of smooth muscles surrounding the epididymis and sexual glands, which allows the release of sperm cells and glandular secretions; and the ejaculatory reflex, which determines rhythmic contractions of the striated bulbospongiosus, ischiocavernosus, and urethral muscles, with consequent expulsion of ejaculate (13). Defects in these events, such as obstruction of the efferent ducts or retrograde ejaculation, can cause azoospermia (10). In the literature, a few cases of ampullae obstruction, or plugged ampullae, in equine (14) and donkey stallions (15) are reported. Furthermore, a segmental aplasia in a stallion has been described, which caused azoospermia (16), and a case of retrograde ejaculation, which caused a low-volume ejaculate (13).

The first step to differentiate azoospermia due to abnormalities of spermatogenesis from obstructive azoospermia is to confirm if ejaculation occurred. This can be achieved by detecting rhythmic urethral pulsation, tail flagging, and normal trusting (12, 16, 17). If ejaculatory behavior is normal, alkaline phosphatase from seminal plasma (SPAP) should be assessed (12). SPAP is a dephosphorylating enzyme active in many tissues; in horses, studies suggest that SPAP is mainly produced in the testes and epididymis (14, 18). In these tissues, SPAP activity is relatively high, so in ejaculates complete with seminal fluids, ranges over 1,500 IU/L are reported. In cases of bilateral obstructive azoospermia, instead, lower values (<100 IU/L) are reported, as SPAP activity is low in sex gland tissues (9, 14). In cases of obstructive azoospermia, transrectal palpation and ultrasound can detect ampullae asymmetries, with an enlarged echoic-filled lumen (9, 10). In most cases, obstructive azoospermia is due to an accumulation of spermatozoa in the efferent duct that can create a plug (9). For these aggregates to be expelled, repeated semen collections are necessary. Moreover, administrating 20–30 IU of oxytocin can provide muscular contraction and plug expulsion (11, 15, 16).

This report describes the case of a 20-year-old Murgese stallion diagnosed with obstructive azoospermia. To the best of the authors’ knowledge, this report represents a unique, as hyaline deposits were detected in ampullae glandular ducts and corpora amylacea in vesicular gland tissue. Moreover, in this report, endoscopic treatment was attempted to solve the obstruction.

## 2 Case description

A 20-year-old Murgese stallion was referred for acquired azoospermia, evidenced during regular breeding soundness evaluation before the breeding season. The patient regularly underwent vaccinations and was tested negative for sexually transmitted diseases. History was negative for reproductive disorders, as the animal previously underwent regular reproductive evaluation with positive results and was successfully involved in breeding programs.

**Figure 1:**
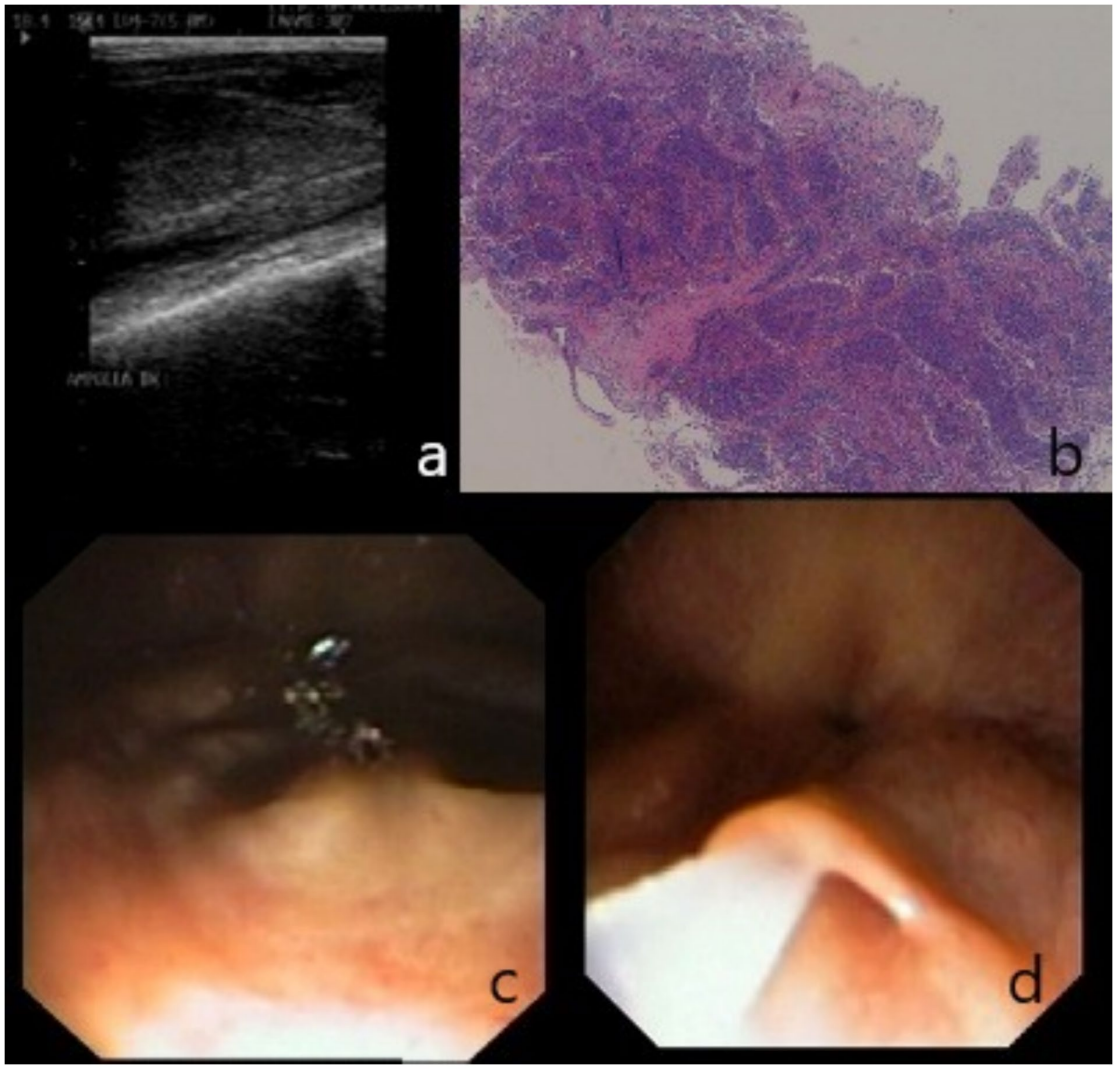
Findings of clinical examination: **(a)** ultrasonographic visualization of right ampulla; **(b)** testicular biopsy, spermatogenesis is evident in seminiferous tubules; **(c)** endoscopic visualization of the urethral opening of ampullae. **(d)** Detail of ampullae cannulation and insufflation.

General clinical examination and evaluation of internal and external genitalia did not reveal significant alterations. No valuable anomalies were detected on palpation of the glands. The ultrasound examination was also negative for neoformations or degeneration (Figure 1a). Semen collection was then performed twice, one hour apart: mating behavior and libido were regular for the species; also, during semen collection, the operator correctly detected rhythmic urethral pulsations and tail flagging. The volume obtained was adequate (80 mL after first collection and 50 after the second), but the ejaculate was transparent, and further microscopic evaluation highlighted the absence of spermatozoa in both samples.

Two days later, the stallion underwent bladder catheterization before and after semen collection; the urinalysis was normal and negative for spermatozoa. An alkaline phosphatase from seminal plasma (SPAP) assay was then performed on the seminal plasma, returning a value of 30 IU/L, consistent with an obstruction of the efferent ducts (14). Testicular tru-cut biopsy was also performed (19), revealing, in the parenchyma of both testicles, the presence of an adequate number of seminiferous tubules with normal architecture, within which normal spermatogenesis was detected (Figure 1b).

## 3 Treatment and follow-up

Attempts were made to obtain the emission of sperm by performing a transrectal massage of the ampullae and administering 20 IU of oxytocin intravenously (11, 16), then collecting semen twice, one hour apart, every other day, for a week, with negative results.

An endoscopic resolution was then attempted. The horse, adequately restraint in a stock, was sedated intravenously using 0.03 mg/kg of acepromazine (Prequillan, Fatro, Italy) and 0.1 mg/kg of romifidine (Sedivet, Boehringer Ingelheim, Italy). Anesthesia for the standing procedure was maintained with a constant rate infusion of 0.03 mg/kg/h of romifidine. An 8 mm flexible videoendoscope was gently inserted into the urethra following a thorough cleaning of the penis. Air insufflation promoted the correct sliding of the endoscope and allowed the seminal colliculus, the opening of bulbourethral glands, vesicular glands, and ampullae to be visualized (Figure 1c). Ampullae were gently insufflated (Figure 1d), and a further transrectal massage was performed, followed by the emission of glandular secretions.

No adverse effects, due to the procedure or the anesthetic protocol, were detected after endoscopy; in the following days, however, several attempts to collect semen did not result in the emission of a complete ejaculate. Therefore, the horse was declared functionally sterile and excluded from breeding, also considering the advanced age, without performing further diagnostic investigations or treatment attempts.

Approximately 5 years after being diagnosed with occlusive azoospermia, the stallion died of natural causes at the age of 25, having shown no clinical signs attributable to other systemic or reproductive system pathologies, and was thereafter necropsied (Table 1).

**Table 1:** Case report timeline.

	Clinical procedures	Findings
1	Clinical evaluation	No testicular alteration or abnormalities upon transrectal palpation
2	Semen collection	Adequate libido and volume. No sperm detected (azoospermia)
3	Urethral catheterization	Negative urinalyses
4	Semen collection for SPAP assay	SPAP = 30 IU/L (ranges over 1,500 IU/L)
5	Testicular biopsy	complete germ lines in both testes
6	20 UI oxytocin administration	No sperm in the ejaculate
7	Endoscopy and transrectal massage	No resolution. Exclusion of the stallion from reproduction
8	Death for natural causes and necropsy	Sexual glands enlargement
9	Histopathological investigation	Spermatogenesis present in both testicles, but degenerative changes evident. Distended glandular lumens with concentric lamellar concretions, referable to corpora amilacea (vesicular glands). Ectasic lumen, filled with hyaline substance, occasionally showing concentric stratification (ampullae).

**Figure 2:**
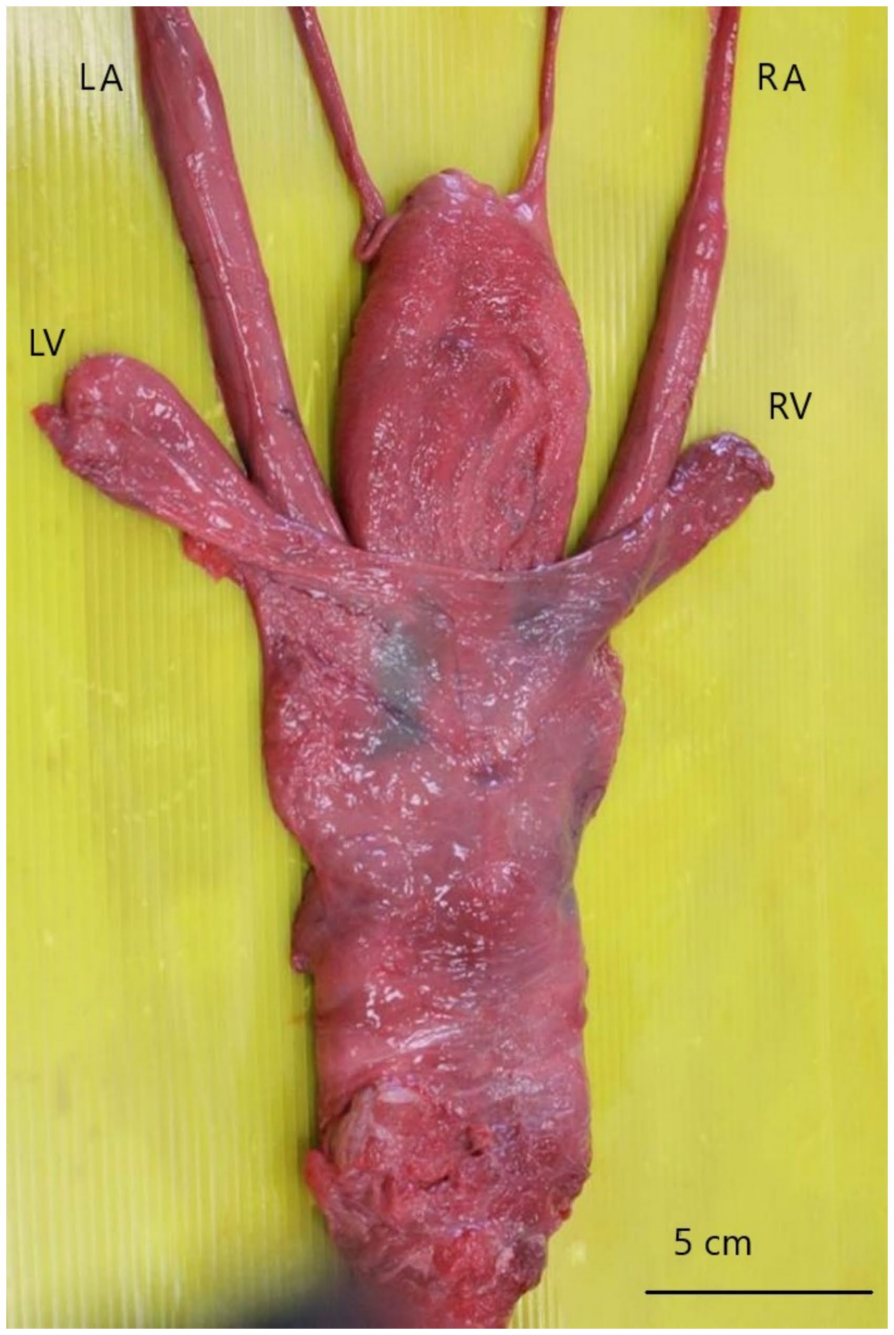
Accessory sexual glands, dorsal view. The left ampulla (LA) and left vesicular gland (LV) are larger than the opposite ones (RA; RV).

At necropsy, gross inspection of the entire genital tract revealed a prominent enlargement of the left vesicular gland, which contained a sandy-like substance. Likewise, the left ampulla appeared moderately enlarged (Figure 2).

Representative tissue samples were collected from testes, vesicular glands, ampullae, promptly fixed in 10% neutral buffered formalin, and routinely processed for histopathological investigations (hematoxylin and eosin stain).

**Figure 3:**
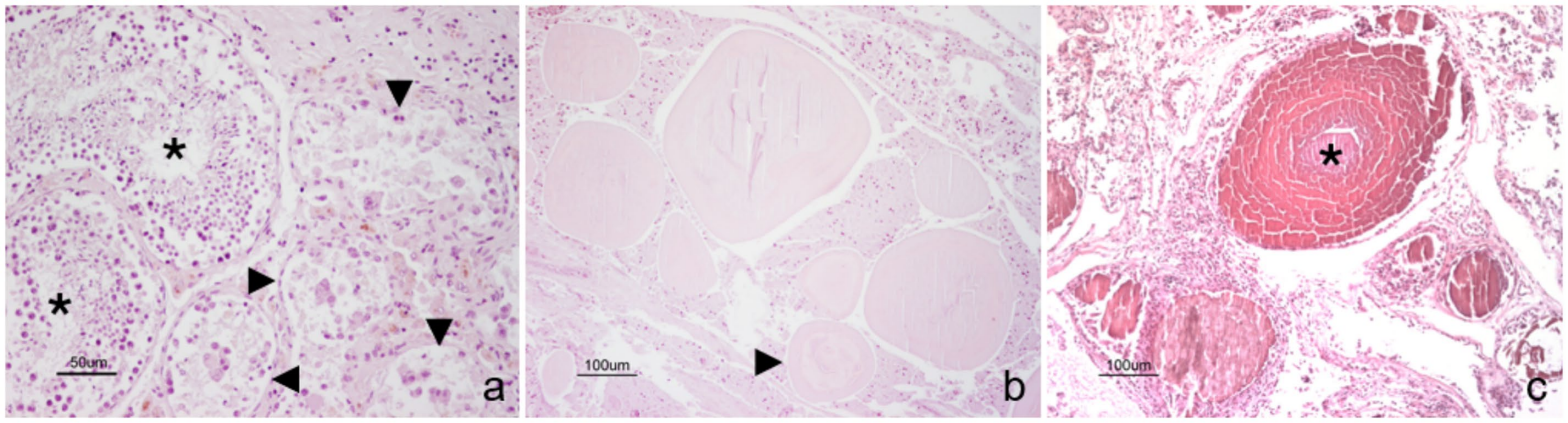
Stallion. Genital tract. **(a)** Spermatogenesis is evident in seminiferous tubules (black asterisks), although degenerative changes are also present in contiguous tubules (black arrowheads). **(b)** Ampullary glands are filled and distended by hyaline substance, which occasionally show a lamellar concentric appearance (black arrowhead). **(c)** Likewise, the lumina of seminal vesicles appear ectasic and filled with mineralized material, which often shows a prominent lamellar structure (black asterisk). Hematoxylin and eosin stain. Final magnification: x200 **(a)**, x100 **(b,c)**.

Microscopically, spermatogenesis was still present in both testicles, although degenerative changes were evident within several seminiferous tubules (Figure 3a). Interstitial Leydig cells were clearly detectable and often appeared lipofuscin laden. Moreover, peritubular neutrophilic infiltrates were focally seen.

All the glandular lumens of the left vesicular gland were distended and filled with mineralized concretions, often showing a concentric lamellar appearance, morphologically referable to corpora amylacea. Similar, although less severe findings were observed in the right vesicular gland (Figure 3b).

The lumen of both the ampullae was ectasic, while the ampullary ducts were distended and filled with hyaline substance, occasionally showing concentric stratifications with initial evidence of mineralization (Figure 3c).

## 4 Discussion and conclusion

Azoospermia is a rarely reported condition in stallions, and only a few cases are truly azoospermic (seminal fluid devoid of sperm). In most cases, azoospermia is related to ejaculation failure or dysfunction (11, 12). Cases of obstructive azoospermia are reported both in horses and donkeys (14–16), but also in other species such as boars (20), and men (21, 22). In some stallions, abnormal semen retention in the efferent ducts has been identified. In these cases, ejaculates are highly concentrated, with low motility, and occasionally, sperm can form a plug, retained in the distal efferent duct (ampulla), causing a mono or bilateral occlusion (9). This condition is therefore acquired and can be readily detected by performing regular reproductive evaluations of stud horses. Interestingly, also in the present case, acquired azoospermia due to occlusion of the deferent ducts was highlighted, probably due to a non-responsive abnormal sperm retention. Regular BSE allowed for rapid identification of the newly emerging problem. In fact, in previous breeding seasons, sperm analysis was always within normal ranges for the species. History is of paramount importance in BSE because it allows discrimination of new-onset problems from pre-existing ones (6, 8). In this case, in addition to the semen analysis, it was known that the subject was of proven fertility, had numerous offspring, and was tested negative for infectious sexual diseases. Similar findings are described by Turner and McDonnell (14), reporting a sudden decrease in fertility, and azoospermia in an 11-year-old stallion, subsequently diagnosed with bilaterally blocked ampullae. Segabinazzi et al. (15) report different findings: in their case, a progressive decrease in fertility and sperm parameters is described in a jack in his first breeding season and with previous good semen evaluation. In this case, spermiostasis was suspected (15). In the case described by Estrada (16) instead, history highlighted previous infertility, as the stallion was diagnosed with a congenital defect. As for clinical evaluation and ultrasonography, it must be noted that in the reported case, no ampullar or vesicular gland abnormalities were detected at the time of diagnosis, even if, in gross anatomy, a marked asymmetry was highlighted. We suspect that, at the time of diagnosis, the occlusion was due to semen retention, and a slight variation could have been unnoticed by transrectal palpation or ultrasound. Generally, ampullary occlusion can be readily noticed upon ultrasound imaging, which may detect luminal ectasia (9). However, it should be emphasized that, as reported by Pozor and McDonnell (23), the echogenic characteristics of the sexual glands vary greatly between stallions and are also influenced by sexual activity. Moreover, in cases of semen retention, clinical findings can be mild to absent (24). Semen collection and evaluation are mandatory in cases of azoospermia, primarily to confirm the correct and complete ejaculation. In the present report, secondary signs, such as flag tail and urethral pulsation (2, 12, 15–17) have been detected, thus confirming the complete ejaculation. This evidence, with the concurrence of azoospermia, was highly indicative of an obstruction; for this reason, seminal alkaline phosphatase was assessed. A higher activity of this enzyme is reported in testicular and epididymal tissue, than in glandular tissue (14), so low values, as detected in the present case, indicate the absence of testicular secretion, thus confirming obstructive azoospermia. Blanchard et al. (12) also suggests ruling out retrograde ejaculation as in this condition alkaline phosphatase is low. Urinalysis was also performed in the present report and detected no sperm. However, it should be noted that in cases of retrograde ejaculation, azoospermia is not the most common finding, but generally low ejaculate volume is detected (12, 13). As for testicular biopsies, this procedure is not strictly necessary when evaluating occlusive azoospermia. It is generally applied for azoospermia due to testicular degeneration (17) or blockage of spermatogenesis due to other causes (e.g., hormonal alterations) (12). Nevertheless, in these circumstances the SPAP in the ejaculate is usually high, unlike the case in question, in which values below the cutoff of 100 IU/L were found (9). However, the authors decided to perform this procedure anyway since, due to the age of the stallion, testicular degeneration was also initially suspected. Different techniques are described for performing testicular biopsies: open biopsies, punch, or tru-cut (25, 26), the latter being less invasive than the open technique. The authors used the tru-cut technique, as it has been demonstrated that using an 18 G needle determines minimal tissue damage, resulting in a hemorrhagic suffusion that resolves in 7 days (19). Although it has been demonstrated in recent work that the effects of biopsies, even repeated, are minimal and short-term, this procedure is sometimes avoided due to the risks of adverse effects such as hemorrhages or adhesions (27). An alternative but equally valid option is fine needle aspiration (FNA), which allows for adequate cellularity for diagnosis with a less invasive procedure (25). However, the authors preferred tru-cut biopsy, and the procedure reported no adverse effects on the patient. Furthermore, the testicular biopsy showed normal spermatogenesis and no signs of degeneration, as found in post-mortem histopathology.

The initial therapeutic approach to obstruction was the administration of oxytocin, associated with repeated transrectal massages of the ampullae and consecutive semen collection in line with what is described in the literature (11, 15, 16). These procedures should facilitate the expulsion of any sperm plugs (9) or solve sperm retention, but in the present case, they were not effective. For this reason, the authors attempted an endoscopic approach to cannulate the opening of the ampullae and unblock them with a delicate insufflation, with negative results. To the authors’ knowledge, no similar approaches are described, although Estrada et al. (16) performed endoscopy to determine the anatomical alteration detected upon palpation. This technique is however indicated as a collateral investigation even in case of obstruction, allowing the glands to be visualized, as in the case presented, even if the ampullae are not readily cannulated (9, 28). Regarding the anesthetic protocol used for endoscopy, it must be noted that the use of acepromazine is generally not recommended as, although it improves penile protrusion, can increase the risk of penile paralysis and paraphimosis (29). However, in the authors’ experience, the use of low doses of acepromazine, as in the present case, determines penile extension but is not associated with the risk of paraphimosis. In the literature, a protocol with acepromazine up to 0.06 mg/kg is also reported (30), a double dosage compared to that used in this report.

At the time of the animal’s death, unfortunately, a new reproductive evaluation was not carried out, and, therefore, it is not possible to say whether the marked asymmetry of the glands, found on gross anatomy, could have been detected through palpation and ultrasound. It is interesting to note how, upon dissection, the contents of the glands, particularly the vesicular glands, had a granular appearance, like sand, probably as a result of the deposition of secretions. To the authors’ knowledge, nothing similar has been described for the equine stallion. The histological examination then highlighted deposits of protein material occluding the glandular lumens of the ampullae of the deferens and the vesicular glands, with calcareous deposits and lamellar structure in the latter, which gave them the typical appearance of corpora amylacea. Morgani described corpora amylacea (CA) for the first time in 1779 as concretions derived from the pathological precipitation of prostatic secretions that stained with iodine (31). CA are laminated luminal secretions commonly present in human prostatic glands and increase with aging (32). Recent studies also showed that in men the presence of CA is correlated with prostate cancer, therefore, when identified, they are indicative for carrying out further investigations (33). CA exhibit a lamellar structure, contain amyloid material, and have been isolated also in other human tissues, such as the nervous system and muscles (30). Although many similarities are found between human and canine prostatic pathologies, CAs are rarely observed in the canine prostate and generally have characteristic concentrical lamellations, can calcify, and are PAS-positive. Even in dogs, CA seem to be related to aging (34). Based on the literature and to the best of the authors’ knowledge, no cases of corpora amylacea in horse sexual glands are described. The authors hypothesize that this finding could be, as for the canine species, a rare occurrence and, as for humans, the corpora amylacea could represent precipitates of glandular secretions, related to aging, in physiological conditions. The correlation between these abnormally diffused intraductal deposits and sperm retention/ampullary obstruction remains uncertain, but the authors hypothesize that abnormal glandular obstruction could be a consequence of ejaculatory dysfunction. The lesions would appear to be coeval; however, the different levels of mineralization, more advanced in the vesicular glands and which led to the formation of the CA, may be due to the different pH of the secretions.

In conclusion, this report aimed to describe not only a correct and complete diagnostic approach to obstructive azoospermia in the stallion but also describes, for the first time, the presence of corpora amylacea in the vesicular glands of the stallion, a condition that could be age-related, also in normal circumstance. Further studies are required to confirm this fascinating hypothesis.

## Data Availability

The original contributions presented in the study are included in the article/supplementary material, further inquiries can be directed to the corresponding author.

## References

[1] Kenney RM. Clinical fertility evaluation of the stallion. Ann Meet Am Assoc Equine Practiti. (1976):336–55.

[2] Whitesell K Stefanovski D McDonnell S Turner R. Evaluation of the effect of laboratory methods on semen analysis and breeding soundness examination (BSE) classification in stallions. Theriogenology. (2020) 142:67–76. doi: 10.1016/j.theriogenology.2019.09.035, PMID: 31581045

[3] Purswell BJ Althouse GC Root Kustritz MV Pretzer S Lopate C. Guidelines for using the canine breeding soundness evaluation form. Theriogenol Handbook. (1992) 51–59. SA-C1 (8/92)

[4] Wolfe DF. Abnormalities of the bull–occurrence, diagnosis, and treatment of abnormalities of the bull, including structural soundness. Animal. (2018) 12:S148–57. doi: 10.1017/S1751731118000939, PMID: 29717682

[5] Johnson AK. Normal feline reproduction: the tom. J Feline Med Surg. (2022) 24:212–20. doi: 10.1177/1098612X221079707, PMID: 35209771 PMC10845405

[6] Crabtree J. Prebreeding examination of the stallion: 1. Phys Examination Pract. (2010) 32:22–8. doi: 10.1136/inp.b5503

[7] Hurtgen JP. Evaluation of the stallion for breeding soundness. Vet Clin N Am Equine Pract. (1992) 8:149–65. doi: 10.1016/S0749-0739(17)30472-8 1576547

[8] Varner DD. Approaches to breeding soundness examination and interpretation of results. J Equine Vet Sci. (2016) 43:S37–44. doi: 10.1016/j.jevs.2016.06.075

[9] Ball BA. Diagnostic methods for evaluation of stallion subfertility: a review. J Equine Vet Sci. (2008) 28:650–65. doi: 10.1016/j.jevs.2008.10.003

[10] Varner DD Blanchard TL Brinsko SP Love CC Taylor TS Johnson L. Techniques for evaluating selected reproductive disorders of stallions. Anim Reprod Sci. (2000) 60-61:493–509. doi: 10.1016/S0378-4320(00)00115-9, PMID: 10844219

[11] McDonnell SM. Ejaculation: physiology and dysfunction. Vet Clin N Am Equine Pract. (1992) 8:57–70. doi: 10.1016/S0749-0739(17)30466-2, PMID: 1576554

[12] Blanchard TL Brinsko SP Varner DD Love CC. How to investigate azoospermia in stallions. Proc. Am. Ass. Equine Practnrs. (2009) 55:331–5. doi: 10.5555/20103149546

[13] Brinsko SP. Retrograde ejaculation in a stallion. J Am Vet Med Assoc. (2001) 218:551–3. doi: 10.2460/javma.2001.218.551, PMID: 11229508

[14] Turner RMO McDonnell SM. Alkaline phosphatase in stallion semen: characterization and clinical applications. Theriogenology. (2003) 60:1–10. doi: 10.1016/S0093-691X(02)00956-1, PMID: 12620574

[15] Segabinazzi LG Silva LF Okada C Medrado F Papa F Alvarenga MA. Plugged ampullae in a donkey stallion (*Equus asinus*). J Equine Vet Sci. (2018) 63:24–6. doi: 10.1016/j.jevs.2017.12.012

[16] Estrada A Samper JC Lillich JD Rathi RR Brault LS Albrecht BB. Azoospermia associated with bilateral segmental aplasia of the ductus deferens in a stallion. J Am Vet Med Assoc. (2003) 222:1740–3. doi: 10.2460/javma.2003.222.1740, PMID: 12830868

[17] Gehlen H Bartmann CP Klug E Schoon HA. Azoospermia due to testicular degeneration in a breeding stallion. J Equine Vet Sci. (2001) 21:137–9. doi: 10.1016/S0737-0806(01)70110-4

[18] Bucci D Giaretta E Spinaci M Rizzato G Isani G Mislei B. Characterization of alkaline phosphatase activity in seminal plasma and in fresh and frozen–thawed stallion spermatozoa. Theriogenology. (2016) 85:288–295.e2. doi: 10.1016/j.theriogenology.2015.09.007, PMID: 26433714

[19] Carluccio A Zedda MT Schiaffino GM Pirino S Pau S. Evaluations of testicular biopsy by tru-cut in the stallion. Vet Res Commun. (2003) 27:211–3. doi: 10.1023/B:VERC.0000014142.20416.80, PMID: 14535392

[20] Clements KM Shipley CF Coleman DA Ehrhart EJ Haschek WM Clark SG. Azoospermia in an 8-month-old boar due to bilateral obstruction at the testis/epididymis interface. Can Vet J. (2010) 51:1130–4. Available at: https://pmc.ncbi.nlm.nih.gov/articles PMID: 21197205 PMC2942052

[21] Ball RY Mitchinson MJ. Obstructive lesions of the genital tract in men. Reproduction. (1984) 70:667–73. doi: 10.1530/jrf.0.0700667 6699820

[22] Buffat C Patrat C Merlet F Guibert J Epelboin S Thiounn N. ICSI outcomes in obstructive azoospermia: influence of the origin of surgically retrieved spermatozoa and the cause of obstruction. Hum Reprod. (2006) 21:1018–24. doi: 10.1093/humrep/dei418, PMID: 16361290

[23] Pozor MA McDonnell SM. Ultrasonographic measurements of accessory sex glands, ampullae, and urethra of normal stallions of various size types. Theriogenology. (2002) 58:1425–33. doi: 10.1016/S0093-691X(02)01034-8, PMID: 12387354

[24] Turner RM. How to perform an examination of the internal reproductive tract of the stallion. Proc. Am. Ass. Equine Practnrs. (2014) 60:294–301. doi: 10.5555/20153416465

[25] Leme DP Papa FO. How to perform and interpret testicular fine needle aspiration in stallions. J Equine Vet Sci. (2010) 30:590–6. doi: 10.1016/j.jevs.2010.09.003

[26] Pearson LK Rodriguez JS Tibary A. How to obtain a stallion testicular biopsy using a spring-loaded split-needle biopsy instrument. Proc. Am. Ass. Equine Practnrs. (2011) 57:219–25. doi: 10.5555/20123295090

[27] Rode K Sieme H Otzen H Schwennen C Lüpke M Richterich P. Effects of repeated testicular biopsies in adult warmblood stallions and their diagnostic potential. J Equine Vet Sci. (2016) 38:33–47. doi: 10.1016/j.jevs.2016.01.003

[28] McCue PM. Endoscopic examination of the urethra In: Dascanio J. McCue P. M., editors. Equine reproductive procedures, Wiley Blakwell (2014). 458–60. doi: 10.1002/9781118904398.ch140

[29] Menzies-Gow N. Diagnostic endoscopy of the urinary tract of the horse. In Pract. (2007) 29:208–13. doi: 10.1136/inpract.29.4.208

[30] Bertone JJ. Urinary tract endoscopy of horses. Proc Am Ass Equine Practnrs. (1998) 44:298–9.

[31] Riba M Del Valle J Augé E Vilaplana J Pelegrí C. From corpora amylacea to wasteosomes: history and perspectives. Ageing Res Rev. (2021) 72:101484. doi: 10.1016/j.arr.2021.101484, PMID: 34634491

[32] Kaplan KJ. Nebulous corpora amylacea. Arch Pathol Lab Med. (2005) 129:543–3. doi: 10.5858/2005-129-543-NCA, PMID: 15794686

[33] Palangmonthip W Wu R Tarima S Bobholz SA LaViolette PS Gallan AJ. Corpora amylacea in benign prostatic acini are associated with concurrent, predominantly low-grade cancer. Prostate. (2020) 80:687–97. doi: 10.1002/pros.23980, PMID: 32271960 PMC10561550

[34] Palmieri C Fonseca-Alves CE Laufer-Amorim R. A review on canine and feline prostate pathology. Front Vet Sci. (2022) 9:881232. doi: 10.3389/fvets.2022.881232, PMID: 35720846 PMC9201985

